# Lung Microbiome in Lung Cancer: A Systematic Review

**DOI:** 10.3390/microorganisms12122439

**Published:** 2024-11-27

**Authors:** Sergiu-Remus Lucaciu, Bianca Domokos, Ruxandra Puiu, Victoria Ruta, Stefania Nicoleta Motoc, Ruxandra Rajnoveanu, Doina Todea, Anca Mirela Stoia, Adina Milena Man

**Affiliations:** Department of Pneumology, Iuliu Hațieganu University of Medicine and Pharmacy, 400012 Cluj-Napoca, Romania; lucaciuserju@gmail.com (S.-R.L.); ruxi.puiu@yahoo.com (R.P.); victoria.suteu@yahoo.com (V.R.); motoc_nicoleta@yahoo.com (S.N.M.); ruxandra.rajnoveanu@umfcluj.ro (R.R.); dtodea@umfcluj.ro (D.T.); mirelastoia@yahoo.com (A.M.S.); manmilena50@yahoo.com (A.M.M.)

**Keywords:** lung microbiome, lung cancer

## Abstract

To date, the percentage composition of the lung microbiome in bronchopulmonary cancer has not been summarized. Existing studies on identifying the lung microbiome in bronchopulmonary cancer through 16S rRNA sequencing have shown variable results regarding the abundance of bacterial taxa. Objective: To identify the predominant bacterial taxa at the phylum and genus levels in bronchopulmonary cancer using samples collected through bronchoalveolar lavage and to determine a potential proportional pattern that could contribute to the diagnosis of bronchopulmonary cancer. Data Sources: A systematic review of English articles using MEDLINE, Embase, and Web of Science. Search terms included lung microbiome, lung cancer, and bronchoalveolar lavage. Study Selection: Studies that investigated the lung microbiome in bronchopulmonary cancer with samples collected via bronchoalveolar lavage. Data Extraction: Independent extraction of articles using predefined data fields, including study quality indicators. Data Synthesis: Nine studies met the inclusion criteria, focusing on those that utilized a percentage expression of the microbiome at the phylum or genus level. There was noted heterogeneity between studies, both in terms of phylum and genus, with a relatively constant percentage of the *Firmicutes* phylum and the genera *Streptococcus* and *Veillonella* being mentioned. Significant differences were also observed regarding the inclusion criteria for study participants, the method of sample collection, and data processing. Conclusions: To date, there is no consistent percentage pattern at the phylum or genus level in bronchopulmonary cancer, with the predominance of a phylum or genus varying across different patient cohorts, resulting in non-overlapping outcomes.

## 1. Introduction

Lung cancer stands as the second most frequently diagnosed cancer and held the grim distinction of being the primary cause of cancer-related deaths in 2020. It accounted for about one in 10 (11.4%) of all diagnosed cancers and a staggering one in 5 (18.0%) of all cancer-related deaths. Notably, lung cancer is the foremost contributor to both cancer morbidity and mortality among men. In contrast, among women, it ranks third in terms of incidence, following breast and colorectal cancer, but rises to the second position for mortality, surpassed only by breast cancer [1].

The human microbiota encompasses 10–100 trillion symbiotic microbial cells hosted by each individual, predominantly bacteria located in the gut. Clarifying the definition of the human microbiome has been challenging due to confusion surrounding terminology. For instance, “microbiota” is frequently used to refer to the microbial taxa associated with humans, while “microbiome” is used interchangeably but specifically denotes the catalog of these microbes and their genes. Initially, “metagenomics” described the shotgun analysis of total DNA. However, its current usage is expanding to include investigations of marker genes like the 16S rRNA gene. What sets today apart is not just the capacity to observe evident distinctions, but rather the capability to employ advanced molecular techniques to comprehend the reasons behind these differences. This enables us to gain insights into why variations exist and understand the mechanisms through which transformations from one state to another can be influenced. Scientists can now produce millions of sequences per sample, enabling the examination of variations in microbial communities across body sites and among individuals [2].

The composition of the adult microbiome is shaped by a combination of factors such as host genetics, diet, environmental influences, and external factors like antibiotic usage, which has the potential to disrupt the balance in the gut. Any significant alteration in microbial composition can adversely impact the human host, potentially playing a role in the initiation of various conditions, including cancer. In contrast to the microbe-friendly conditions found in the gut, oral cavity, and upper airways, the lower airway seems to present an inhospitable environment, characterized by limited nutrients and high oxygen stress, creating challenges for microbial survival. Drawing on metaphors of adapted island models, one could liken the lower airway to a desert in terms of microbial colonization and replication. While it was traditionally believed to be sterile, emerging evidence now reveals a diverse array of microbial species in the lungs of healthy individuals. These species are predominantly anaerobes, including Gram-negative *Prevotella* and *Veillonella* spp., as well as Gram-positive *Coprococcus* and *Dorea* spp. [3].

Similar to any mucosal surface in the human body, the host immune system is not oblivious to microbial exposures. In fact, the microbial products present in the lower airways result from a delicate equilibrium between the influx of microbes and the removal of microbes through clearance mechanisms [4].

The microbial composition and biomass in the upper and lower respiratory tracts differ significantly. In the upper respiratory tract, which includes the nasal cavity, paranasal sinuses, pharynx, and supraglottic portion of the larynx, bacteria are prevalent. Additionally, there are topographical variations in microbial composition within the upper respiratory tract. For instance, the dominant taxa in the nasal cavity and nasopharynx consist of *Moraxella*, *Staphylococcus*, *Corynebacterium*, *Haemophilus*, and *Streptococcus* species. In contrast, the oropharynx shows a high abundance of *Prevotella, Veillonella, Streptococcus, Leptotrichia*, *Rothia*, *Neisseria*, and *Haemophilus* species [5]. On the other hand, the lower respiratory tract, which includes the trachea and lungs, maintains a relatively low microbial biomass. It plays a major role in lower airway mucosal immunology. This low biomass is upheld by rapid microbial clearance through various physiological mechanisms. These mechanisms facilitate the lower respiratory tract in performing its crucial function: the exchange of oxygen and carbon dioxide [3,4].

The concentration of bacterial DNA in the lungs is approximately 100 times lower than that found in the oral cavity. Healthy lungs are consistently exposed to oropharyngeal bacteria through subclinical aspiration, a process confirmed both radiographically and ecologically in healthy, asymptomatic individuals [6].

In healthy lungs, the microbial biomass is significantly lower, ranging from 10^3^ to 10^5^ bacteria per gram of tissue [7], compared to the much higher microbial density in the lower gastrointestinal (GI) tract, which typically ranges from 10^11^ to 10^12^ bacteria per gram of tissue [8]. Despite the shared embryological origin of the GI tract and lungs as mucosa-lined luminal organs, their micro-anatomical features exhibit notable differences. This divergence in microbial abundance and anatomical characteristics underscores the distinct environments and functions of these organs within the body. The composition of the lung microbiome is thought to be influenced by the equilibrium of three key factors: the influx of microbes into the airways, the removal of microbes from the airways, and the relative reproduction rates of community members present in the airways. The latter is determined by the regional growth conditions, including factors such as pH, oxygen levels, and nutrient availability [9].

Patients with lung cancer are at a heightened risk of microbial infections. The impact of repeated microbial exposure on reshaping the lung’s immune system has been gaining recognition. Furthermore, the roles of pathogens in lung disease are being rigorously investigated, highlighting the importance of understanding how these interactions may influence disease outcomes. Inflammation resulting from microbial infections is increasingly understood to play a role in the development and progression of cancer [10].

DNA extracted from bacteria is a valuable tool for detecting and identifying microbes in various specimens, regardless of growth conditions. The 16S rRNA gene, with its distinct structure, is a major universal target for bacterial identification. Its nine variable regions and conserved regions allow the design of nearly universal primers, making it widely used in diagnostic departments. However, due to ongoing advancements, there is variability in DNA extraction and analysis processes, and no consensus on a gold standard practice has been established [6].

It is important to name certain terms to better understand the study of the microbiome. Dysbiosis, characterized by a deviation from a normal microbial composition, is linked to various adverse biological phenomena, at times leading to clinical consequences. In the context of the lung, dysbiosis can exert a substantial influence on the initiation and advancement of respiratory diseases, underscoring the clinical imperative to comprehend the biology of the lung microbiome. In the past decade, there has been a swift proliferation of research employing culture-independent genomic techniques to characterize the microbial environment in the lung [4].

Various higher-level metrics are frequently employed to characterize the microbiome within a sample. While these metrics may not offer insights into alterations in the abundance of individual taxa, they enable a more comprehensive assessment of overall changes or distinctions in the microbial composition. Examples of such measures include alpha and beta diversity. Within a single sample, diverse metrics collectively known as alpha diversity are employed to estimate diversity. These metrics encompass aspects such as richness (the number of different taxa) or distribution (evenness) within a microbial sample, aiming to capture a blend of both properties. Alpha diversity serves as a metric for microbiome diversity within an individual sample, whereas beta diversity gauges the similarity or dissimilarity between two communities. Like alpha diversity, numerous indices exist, each capturing distinct facets of community heterogeneity. These indices differ in how they account for variation in rare species, whether they consider presence/absence alone or incorporate abundance, and how they interpret shared absence. One widely adopted measure, Bray–Curtis dissimilarity, is popular for its consideration of both the size (overall abundance per sample) and shape (abundance of each taxon) of communities (Bray, 1957) [4].

Beta diversity is a term used to quantify how much a community’s membership or structure differs between two samples. A recent examination of taxon-based assessments of beta diversity revealed that certain metrics, like Canberra and Gower distances, possess enhanced capabilities to distinguish clusters. In contrast, other metrics, such as chi-squared and Pearson correlation distances, are better suited to uncovering the impacts of environmental gradients on communities [11].

Various pipelines are available for the analysis of microbial community data, including mothur, WATERS (Workflow for the Alignment, Taxonomy, and Ecology of the Microbial Environment), the RDP (Ribosomal Database Project) pyrosequencing tools, and QIIME (Quantitative Insights Into Microbial Ecology, pronounced “chime”) [12,13,14,15]. QIIME provides a platform for analyzing high-throughput sequencing data, enabling users to import raw sequences and generate metrics for both inter- and intra-sample diversity. Consistent identification of operational taxonomic units (OTUs) and standardized diversity measures are essential in comparing results across studies. However, as sequencing data grows, the OTU concept faces increasing challenges, with phylogenetic approaches gaining more traction [4].

To improve analyses reliant on a limited set of taxonomic names, 16S rRNA sequences can be clustered into operational taxonomic units (OTUs) at 97% similarity (3% difference). This level of clustering is widely accepted for differentiating bacterial organisms below the genus level. However, it is important to recognize that assuming this threshold consistently identifies microbial species or strains can be misleading [16].

LEfSe (linear discriminant analysis effect size) is a tool designed for high-dimensional biomarker discovery, focusing on identifying features such as genes, pathways, or taxa that distinguish between two or more biological conditions. It integrates statistical significance, biological relevance, and effect size to pinpoint features that are not only differentially abundant but also biologically meaningful. By combining standard statistical tests with criteria for biological consistency and relevance, LEfSe effectively identifies the elements most likely to account for differences between studied groups [17].

The primary objective of this study is to identify the predominant bacterial taxa at the phylum and genus levels in the lung microbiome of patients with bronchopulmonary cancer, using data derived from bronchoalveolar lavage samples. Furthermore, this study seeks to investigate the existence of a proportional pattern in the microbial composition that may contribute to a better understanding of the role of the lung microbiome in the diagnosis of bronchopulmonary cancer.

## 2. Material and Methods

The systematic review was conducted to answer the question: “What is the composition of the lung microbiome in bronchopulmonary cancer?” The study was planned, conducted, and reported in accordance with the Preferred Reporting Items for Systematic Reviews and Meta-Analyses (PRISMA) [18].

### 2.1. Search Strategy

We performed a systematic literature search using PubMed (Medline), Cochrane, and Web of Science databases to identify relevant studies published up to 10 November 2023.

The following keywords were used: ((“lung”[MeSH Terms] OR “lung”[All Fields]) AND (“microbiota”[MeSH Terms] OR “microbiota”[All Fields] OR “microbiome”[All Fields]) AND (“lung neoplasms”[MeSH Terms] OR (“lung”[All Fields] AND “neoplasms”[All Fields]) OR “lung neoplasms”[All Fields] OR (“lung”[All Fields] AND “cancer”[All Fields]) OR “lung cancer”[All Fields]) AND (“bronchoalveolar lavage”[MeSH Terms] OR (“bronchoalveolar”[All Fields] AND “lavage”[All Fields]) OR “bronchoalveolar lavage”[All Fields])) AND (medline)).

### 2.2. Studies Selection and Eligibility Criteria

Studies that analyzed the lung microbiome in bronchopulmonary cancer were included. The studies had to employ bronchoalveolar lavage as the method of sample collection, and the data processing technique must have been 16S rRNA sequencing. These studies should have expressed their results as percentages, especially at the phyla and genera levels. Articles written in English were considered. The study selection ranged from the year 2007 until 10 November 2023. Studies that evaluated the microbiome through sputum examination or postoperative assessments were excluded. Patients of any age, with any smoking status, either with a prior confirmed diagnosis of cancer before sample collection or with a suspicion of cancer confirmed later, were included.

### 2.3. Study Objectives

The primary objective was to identify the composition and ratio of the lung microbiome in bronchopulmonary cancer, with an assessment of its potential role in the diagnosis of bronchopulmonary cancer. Secondary objectives included evaluating methods of sample collection, processing, and assessing potential compositional differences based on these methods.

### 2.4. Data Extraction and Synthesis

Study characteristics (first author, country, number of patients, sample data, analytical method, main result) were extracted from the included articles and summarized in Table 1. Data extraction was performed by one author (L.S) and independently reviewed by an additional author.

## 3. Results

### 3.1. Literature Search

**Figure 1:**
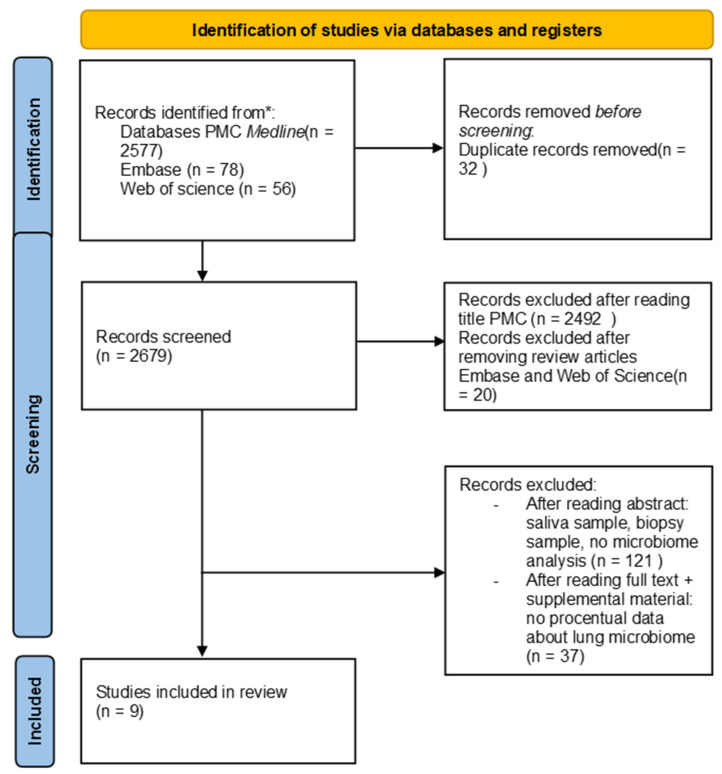
Flow diagram.

Out of 2711 studies identified in the search, 32 were excluded after removing duplicates, and 20 were excluded after reviewing the reviews (Web of Science and Embase). Additionally, 2492 studies from PMC were excluded after title screening, leaving 167 articles. After reviewing the abstracts, studies that were not relevant to the question of the systematic review were excluded, resulting in a smaller selection. Upon further examination of the full text and supplementary materials, additional studies were removed for lacking microbiome percentage data. Overall, nine studies were selected for inclusion in the systematic review (see Figure 1).

### 3.2. Characteristics of the Included Studies

#### 3.2.1. Studies Objective

One study compared the microbiota in saliva, BAL (obtained directly from the excised lobe), non-malignant, peritumoral, and tumor tissue from 18 NSCLC patients eligible for surgical treatment. Bronchoalveolar lavage was performed on 15 patients [21]. Another study, which conducted BAL on 47 patients, compared the differences in microbiota diversity in the oral cavity and bronchoalveolar lavage fluid (BALF) of patients with lung cancer and healthy subjects [27].

Zeng W. et al. (2022) investigated the differences in microbiota composition and gene expression between benign lung disease and non-small cell lung cancer [28]. In another study involving 84 patients, the variations in lung microbiomes between lung cancer patients were analyzed [23]. Another study investigated the association of the microbiota with lung cancer [24]. In Portugal, two studies were conducted, each including 49 lung cancer patients who underwent BAL; one compared the microbiota in lung cancer vs. controls [20], and the other compared the microbiota in lung cancer vs. other lung diseases [25]. In South Korea, a study including 20 lung cancer patients who underwent BAL was used to characterize and compare the microbiomes of lung cancer patients with those having benign mass-like lesions [19].

Liu B. et al. (2022) examined the characteristics of lung microbiota and metabolites in patients, identifying potential biomarkers for lung cancer diagnosis [26]. In a study involving 84 patients, differences in lung microbiomes based on histological types of lung cancer were compared [28]. The V3–V4 regions of the 16S rRNA were most frequently used for the identification and classification of microorganisms, likely due to their specificity and variability [21,23,24,26,28]. In one study, the V1–V3 regions of the 16S rRNA were used [19], while in two other studies the V4 region was used [25,27]. Gomes et al. used the V3–V4 and V4–V6 regions [23].

#### 3.2.2. Inclusion/Exclusion Criteria

Inclusion and exclusion criteria varied among the studies, with differences likely attributable to the specific objectives of each study.

The primary inclusion criterion in most studies is a diagnosis of lung cancer [19,20,21,22,26,27], although some studies have included patients with suspected lung cancer [23,24,25].

Given the influence of corticosteroids, antibiotics, and immunosuppressive drugs on the pulmonary microbiome, three studies included only patients who had not used corticosteroids or antibiotics prior to microbiome assessment, with exclusion periods of the past month [20], past two months [19], and past three months [26].

An important inclusion criterion was a history of surgical interventions on the respiratory tract, as seen in three studies [19,25,26]. Additionally, three studies included only patients who had not received cancer treatment [19,22,26].

Age was an exclusion criterion in only two studies, with age limits of 18 to 80 years [19] or patients over 20 years [25]. 

#### 3.2.3. Bronchoalveolar Lavage Sample Collection

The techniques used for sample collection varied across all studies. 

Except for Bingula et al., where sampling was performed directly from the excised tissue, bronchoalveolar lavage (BAL) was collected via bronchoscopy [19,20,21,22,23,24,25,26,27].

In studies specifying the volume of fluid collected, a minimum of 30 mL of sterile saline was instilled, with the amount ranging from 30 mL [23,24,25], up to approximately 150 mL (Wang et al. [20]). While local anesthesia or sedation was unnecessary for Bingula’s study due to sampling from resected tissue, of the remaining eight studies, four detailed the anesthesia methods. One study used local anesthesia only, that of Wang et al., while three studies applied both local anesthesia and sedation with midazolam or fentanyl, [21,25,27].

The sample storage conditions varied across studies. In one study, samples were stored at −80 °C until processing [22], they were stored at −70 °C in two other studies [21,27], and at −20 °C initially and then at −80 °C until processing in Seixas’s et al. study, while four studies did not specify storage conditions for the lavage fluid prior to processing [24].

Another factor potentially impacting microbiome composition is the site from which the lavage fluid is collected. In six studies, samples were obtained from the affected lobe or segment ([19,20,21,23,24,27]); in two studies, from the affected and unaffected lung ([22,26]); and in one study, from the contralateral lung [25].

#### 3.2.4. Insights from Reviewed Studies

Bingula R. et al. (2020) confirmed that the pulmonary and oral microbiomes differ in both taxonomy and diversity, and that the tumor’s location in the upper or lower lobes can influence the microbiome. The microbiome was not compared based on the type or stage of cancer. The number of patients was limited. At the phylum level, *Firmicutes* predominated, accounting for 45.7%, followed by Proteobacteria (28%) and *Bacteroidetes* (13.3%). At the genus level, *Pseudomonas* was the most abundant (10.3%), followed by *Blautia* (5.9%) and *Streptococcus* (5.1%) [19].

Wang K. et al. (2019) showed that both in the lung and oral levels, patients with lung cancer had less lung and oral microbiota diversity than healthy controls, and the composition of the microbiome was different in patients with lung cancer compared to healthy subjects. *Pseudomonas* was enriched in patients with adenocarcinoma and small cell lung cancer, while *Veillonella* and *Corynebacterium* were abundant in BAL in patients with squamous carcinoma. *Lactobacillus* was enriched in patients with small cell lung cancer. *Rothia* was found to differ significantly in the adenocarcinoma group. Additionally, *Treponema* and clinical lung cancer markers such as SCCA, CA125, CK-19, CA-199, and CEA were identified in the BALF samples. The phylum-level analysis revealed a predominance of *Firmicutes* 38.42% [20].

In the study comparing the microbiome in patients with low PD-L1 (<10%) versus high PD-L1 (≥10%) group by Jang, H.J. et al. (2021), it was highlighted that the abundances of *Neisseria* and *Veillonella* differed significantly in relation to PD-L1 expression levels and immunotherapy responses. No significant differences in alpha or beta diversity were observed, although *Haemophilus* was predominant in the group of immunotherapy non-responders. Among patients with PD-L1 ≥ 10%, the phylum-level analysis showed *Bacteroidetes* as predominant (39.4%), followed by *Firmicutes* (30.5%) and *Proteobacteria* (6.4%). Conversely, in the PD-L1 < 10% group, *Bacteroidetes* also dominated (39.4%), but *Proteobacteria* was the second most abundant phylum (28.2%), followed by *Firmicutes* (23.2%) [21].

Zhuo M. et al. (2020) showed that there was a difference between the microbiome of the cancerous lung compared to that of the healthy lung in the same patient, and the genera *Spiroplasma* and *Weissella* were significantly enriched in the cancerous lung. The top three dominant phyla, classes, orders, and families were the same in both the healthy and cancerous lungs, as well as the top dominant genera. However, the third at the genera level was *Alloprevotella* in the affected lung and *Prevotella* in the healthy lung. There were no significant differences in terms of alpha diversity and overall composition of the microbiome. They found a greater abundance of phylum *Tenericutes*, as well as its class *Mollicutes* and its genus *Spiroplasma*. In the cancerous lung, the predominant phylum was *Proteobacteria* (34.2%), followed by *Firmicutes* (27.96%) and *Bacteroidetes* (21.46%). Conversely, in the healthy lung, *Proteobacteria* accounted for 32.95%, followed by *Bacteroidetes* (26.65%) and *Firmicutes* (26.46%). At the genus level, the cancerous lung was dominated by *Streptococcus* (10.78%), *Neisseria* (7.54%), and *Alloprevotella* (5.22%), while the healthy lung showed higher proportions of *Streptococcus* (12.04%), *Neisseria* (9.37%), and *Prevotella_7* (7.1%) [22].

Gomes S. et al. (2019) found squamous cell carcinoma cases exhibited greater microbiome diversity compared to adenocarcinoma cases, a finding potentially linked to a heavier smoking history among these patients. In terms of phylum composition, *Proteobacteria* was the most dominant (38.7%), followed by *Firmicutes* (25.4%) and *Actinobacteria* (16.5%). At the genus level, the most abundant were *Haemophilus* (29.5%), *Streptococcus* (10.9%), and *Corynebacterium* (8.2%) [23].

Seixas S. et al. (2021) demonstrated that microbial composition, evenness, and the proportions of *Prevotella* and *Haemophilus* varied significantly among patients with COPD, ILD, and lung cancer. Regarding alpha or beta diversity, no significant differences were found between the non-cancer and lung cancer groups, possibly related to the heterogeneity of the cancer types, with only 34.7% of adenocarcinoma and 10.5% of squamous cell carcinoma subtypes. In the cancer group, *Streptococcus* was significantly increased compared to the non-lung cancer group, and *Prevotella* was increased in the lung cancer group compared to the ILD group. At the phylum level, *Firmicutes* predominated (47.11%), followed by *Proteobacteria* (31.35%) and *Bacteroidetes* (15.52%). At the genus level, the most abundant were *Escherichia/Shigella* (8.80%), *Bacillus* (7.66%), *Streptococcus* (7.45%), *Salmonella* (7.40%), *Staphylococcus* (7.27%), and *Lactobacillus* (6.41%) [24].

Lee S.H. et al. (2016) compared the microbiomes of patients with lung cancer and those with benign mass-like lesions. In patients with lung cancer, an increased presence at the phyla level of *Firmicutes* (*p* = 0.037) and *TM7* (*p* = 0.035) was observed compared to benign tumors. Additionally, at the genera level, a relative abundance was noted for *Veillonella* (*p* = 0.003) and *Megasphaera* (*p* = 0.022), suggesting a potential role in cancer for *Veillonella* and *Megasphaera*. Furthermore, a more complex diversity with higher abundance in α-diversity was noted in patients with cancer. The phylum distribution was dominated by *Bacteroidetes* (39.5%), *Firmicutes* (29.7%), and *Proteobacteria* (22.8%), while at the genus level, *Prevotella* (30.8%), *Neisseria* (13.8%), *Veillonella* (11.4%), *Streptococcus* (10.9%), and *Haemophilus* (7.2%) were predominant [25].

The microbiome composition was different in patients with LC compared to controls, with a decrease in alpha diversity and abundance in *Streptococcus*, *Prevotella*, *Veillonella*, and *Haemophilus* in the study conducted by Liu B. et al. (2022). *Fusobacterium* was also increased in patients with LC compared to controls. The phylum composition was dominated by *Proteobacteria* (45.05%), *Firmicutes* (28.31%), and *Bacteroidota* (14.89%), with the most abundant genera being *Pseudomonas* (35.14%), *Streptococcus* (14.34%), *Prevotella* (9.55%), and *Neisseria* (6.81%) [26].

Jang H.J. et al. (2023) in the study comparing the microbiome based on the histological type of lung cancer highlighted a significant difference between the two groups in terms of α- and β-diversities (*p* = 0.004 for Chao1, *p* = 0.001 for Simpson index, and *p* = 0.011 for PERMANOVA), which were significantly more diverse in patients with adenocarcinoma compared to squamous carcinoma. There was also a significant difference in stages I, II, and IIIA compared to stage IIIB and IV in terms of alpha diversity in patients with NSCLC. In patients with squamous carcinoma, *Actinomyces graevenitzii* was dominant. In patients with adenocarcinoma, populations of *Haemophilus parainfluenza, Neisseria subflava, Porphyromonas endodontalis*, and *Fusobacterium nucleatum* were significantly more abundant compared to squamous carcinoma. At the phylum level, *Bacteroidetes* predominated in adenocarcinoma patients (40.8%), followed by *Proteobacteria* (24.9%) and *Firmicutes* (24.1%). For squamous carcinoma patients, *Bacteroidetes* (35.0%), *Firmicutes* (29.3%), and *Proteobacteria* (27.8%) were the most abundant [27].

### 3.3. Comparative Analysis of Microbial Phyla and Genera Distribution in Lung Cancer

In three of the twelve samples, the dominant phylum was *Firmicutes*. Moreover, in all twelve samples, the percentage of *Firmicutes* exceeded 20%, ranging from 23.2% (with a 95% confidence interval between 0.142 and 0.322) to 47.11% (with a 95% confidence interval between 0.33 and 0.611) [21,24]. In five of the samples, the dominant phylum was *Bacteroidetes*, with the highest percentage being 40.8% (95% confidence interval between 0.3029 and 0.513) [27], while in the rest, it was below 40%, with some studies reporting values as low as 13.3% [11,16]. *Proteobacteria* was the dominant phylum in four samples, with the highest percentage at 45.05% (95% confidence interval between 0.0819 and 0.819), and the lowest at 19.1% (95% confidence interval between 0.107 and 0.275) [14,21].

At the phylum level in the study led by Zeng W. et al., *Firmicutes, Bacteroidetes,* and *Fusobacteriota* were identified as being highly abundant in the lung cancer group [28].

In healthy, non-smoking individuals, the *Bacteroidetes* phylum predominates, accounting for more than 60% of the microbiome composition [29,30,31]. Conversely, in smokers, the dominant phylum is *Actinobacteria*, exceeding 40%, followed by *Proteobacteria* and *Bacteroidetes* [29]. Advanced stages of chronic obstructive pulmonary disease (COPD) are characterized by a shift from the *Bacteroidetes* phylum towards *Proteobacteria*, and sometimes towards *Firmicutes* [32]. In sarcoidosis, the dominant phyla are *Actinobacteria* and *Proteobacteria*, while in interstitial lung disease (ILD), *Proteobacteria* prevails [33]. *Proteobacteria* was identified as one of the predominant bacterial phyla in individuals with asthma [34].

Regarding genus diversity, there was considerable variability observed. In some batches, *Pseudomonas* was found to have the highest percentage, 10.3% [19] and 35.14% [26], whereas in other batches, the percentage was significantly lower [21]. *Streptococcus* had a substantial presence across all batches, ranking within the top five genera in each. The presence of *Veillonella*, suspected in several studies of playing a role in bronchopulmonary cancer, varied between 1.4% [19] and 11.4% [25], being over 4% in most batches, except for in Bingula et al. The presence of *Haemophilus*, known for its role in COPD, showed wide variability across batches, from less than 1% [19], 1.07% [26] to 29.5% [23]. A relatively constant presence in the “top” ranks was the genus *Neisseria*.

In a study, conducted by Jun-Chieh J. Tsay in 2021, several operational taxonomic units (OTUs) identified as belonging to the genera *Veillonella*, *Prevotella*, and *Streptococcus* were found to be enriched in samples from subjects with a worse prognosis [35].

In other analysis of 13 patient samples with non-small cell lung cancer (NSCLC), the genera *Streptococcus*, *Vibrio*, and *Enterobacter* were identified as the most prevalent [10]. In the study conducted by Zeng W. et al., the bacteria *Prevotella*, *Veillonella*, and *Neisseria* were found to be highly abundant in the group of patients with lung cancer [28].

Regarding COPD, besides the increased presence of the genus *Streptococcus*, the significant presence of *Haemophilus* in high percentages was notable. Additionally, in smokers, the percentage of *Haemophilus* is significant [31,36]. In healthy, non-smoking subjects, the predominant genera are *Prevotella*, *Veillonella*, *Actinomyces*, and *Streptococcus* [29,36,37,38]. In patients with sarcoidosis, the genera *Prevotella*, *Streptococcus*, and *Corynebacterium* are significantly present [33,39], while in patients with IPF, the most significant genus is *Streptococcus* (30%), but *Prevotella*, *Veillonella*, or *Staphylococcus* are also noteworthy [33,40]. Pleural fluid from cases of malignant pleural effusion, both from lung cancer and mesothelioma, was found to be enriched with bacteria typically considered to be commensals from oral and gut origins, including genera such as *Rickettsiella*, *Ruminococcus*, *Enterococcus*, and the order *Lactobacillales* [41].

### 3.4. Patient Demographics and Tumor Histology in Selected Studies

Of the nine selected studies, six were conducted on Asian populations, 292 patients, and three on European populations, 113 participants, totaling 405 patients. There were 279 male participants and 133 female participants. In one study, three samples were excluded (2 BALF due to an insufficient number of reads (<1000), and one sample did not specify the sex of the patients whose samples were eliminated. In another study, four samples were excluded because the samples failed to amplify using PCR, without specifying the sex of the patients whose samples were excluded. It should also be noted that likely two studies were conducted on the same patient cohort, totaling 84 patients (Jang H.J. 2021 and 2023). Regarding tumor histology, lung cancer ADK accounted for 218 cases and squamous cell carcinoma for 91 cases and there was 1 large cell carcinoma. Small cell lung cancer accounted for 39 cases, carcinoid for 2 cases, NOS for 10 cases, there were 4 metastases, 2 from colorectal cancer, one from breast cancer, and 1 from renal cancer, and 47 cases were unknown [19,20,21,22,23,24,25,26,27].

### 3.5. Alpha Diversity

Regarding alpha diversity, studies comparing lung cancer with controls found that lung cancer patients had less lung and oral microbiota diversity than healthy controls, and lower abundance in alpha diversity compared with the non-lung cancer group. Among those with lung cancer and benign tumors, it was noted that cancer patients exhibited more complex diversity with higher abundance and α-diversity. Alpha-diversity indices did not vary significantly between LC and non-LC groups [20,24,25,26].

The results regarding the microbiome diversity in patients with squamous carcinoma and adenocarcinoma differ between the study conducted by Jang H.J. et al. and that by Gomes S. et al. [23,27].

No significant difference was noted in terms of alpha diversity in patients with PD-L1 < 10% compared to those with PD-L1 > 10% (*p* = 0.307 for Shannon; *p* = 0.540 for Simpson index). In lung cancer patients, no significant difference in alpha diversity was observed between cancerous and healthy lungs: Shannon (*p* = 0.871) and Simpson diversity index (*p* = 0.627) [21,22].

## 4. Discussion

The results of this study indicate significant variability in the proportional distribution of bacterial taxa at both the phylum and genus levels across the included studies. This variability is substantial and limits the identification of a single predominant taxon or the establishment of a reproducible proportional pattern characteristic of bronchopulmonary cancer. Despite these limitations, an important finding of this study was the identification of potential factors contributing to this variability.

There is considerable variability in the inclusion and exclusion criteria across the studies, indicating tailored patient selection strategies based on the unique objectives and parameters of each study. Most studies primarily included patients with lung cancer, with specific subgroups like NSCLC being a common focus. Common exclusion criteria included recent treatment with antibiotics, corticoids, or other specific medications, underlying conditions like hypertension or diabetes, and previous surgeries or therapies related to the airways [19,20,26]. Most studies conducted to date have involved a small cohort of patients, with varying inclusion/exclusion criteria.

The existence of standardized inclusion and exclusion criteria would significantly contribute to obtaining more efficiently comparable results in microbiome studies. It is crucial to consider the use of antibiotics, as they are known to influence the microbiome. The anti-inflammatory effect of corticosteroids, as well as the potential impact of other medications such as immunosuppressants or probiotics, should also be considered [42]. Developing a comprehensive list of medications that could affect the composition of the pulmonary microbiome, to be restricted prior to sample collection, would be beneficial. The specific duration for which these treatments should be ceased prior to the procedure is another critical aspect. Chronic pathologies that can influence the microbiome composition, such as bronchial asthma, COPD, and ILD, must be taken into account [6,32,42]. The pulmonary microbiome in these conditions has been extensively studied and continues to be a research focus. Additionally, smoking status, including current and former smoking, significantly affects the microbiome composition and should be factored into the study design [29]. These elements could serve as inclusion criteria for studies focusing on patients with these associated pathologies. Conversely, they might also be considered as exclusion criteria to eliminate factors that could further alter the microbiome, such as recent pulmonary infections or autoimmune diseases [43,44]. Age restrictions and health status (e.g., absence of pregnancy, specific BMI range) were also crucial criteria in patient selection, reflecting the need to control for variables that could influence study outcomes.

There is a notable diversity in the sample collection methods across different studies, reflecting tailored approaches based on specific study requirements and patient conditions. Despite variations, common practices include the use of topical anesthesia, saline washes, and immediate freezing of samples for DNA extraction.

For future uniformity of results, it might be beneficial to establish a standard protocol for performing bronchoscopy for lavage purposes. This protocol could include pre-procedural rinsing of the cavity with saline solution, local anesthesia with or without sedation, specification of the volume of saline to be introduced for lavage, and ensuring that the lavage is performed prior to any biopsy procedures, if a biopsy is necessary. Additionally, the collection of a “background sample” is crucial for accurate interpretation of results [38]. Furthermore, it might be valuable to standardize the methods for storing and processing these samples, including specific temperature control requirements and time frames for processing to minimize degradation and ensure sample integrity [45]. Uniform data recording and reporting protocols could also be implemented to facilitate more effective comparison and analysis across different studies.

All included studies used Illumina sequencing platforms (MiSeq or HiSeq) for sample analysis. This choice is probably due to the high accuracy and efficiency these platforms offer in DNA sequencing [19,20,21,22,23,24,25,26,27].

For sample preparation, there were variations in DNA extraction methods and the kits used, such as the FastDNA Spin Kit, PowerSoil DNA Isolation Kit, or kits from Qiagen. This variety suggests that there is no universal standard for DNA extraction from BAL samples, with each laboratory adapting the method according to its specific resources and objectives [21,22,23,24,25].

Additionally, different databases and sequence classification programs (such as QIIME, EzTaxon-e, GenBank, or EzBioCloud) were used for taxonomic analysis, indicating diversity in data interpretation and in the identification of microorganisms present in the samples.

In 2021, a significant revision in the taxonomic classification of prokaryotes was implemented, resulting in the renaming of several well-established phyla to comply with the updated nomenclatural rules. Notable changes include the reclassification of *Firmicutes* as *Bacillota*, *Bacteroidetes* as *Bacteroidota*, *Proteobacteria* as *Pseudomonadota*, and *Actinobacteria* as *Actinomycetota.* In this study, we chose not to replace the original names, considering that the studies included in our review used the previous terminology [46]. 

There is growing interest and an increasing number of studies regarding the pulmonary microbiome in lung cancer, as it may serve as a potential biomarker for diagnosis, monitoring progression, and treatment response in bronchopulmonary cancer. Given that bronchoscopy is frequently utilized in the diagnosis of lung cancer, the collection of bronchoalveolar lavage fluid (BALF) alongside biopsy specimens could represent a promising strategy to enhance predictive capabilities [47].

To date, a pattern indicating the percentage expression of the lung microbiome in bronchopulmonary cancer cannot be utilized.

The continuation of studies on the lung microbiome in bronchopulmonary cancer is necessary, but there is a need for well-defined, universally accepted inclusion/exclusion criteria, similar collection techniques, and proper storage, transport, and processing of samples. Additionally, the databases used, DNA extraction techniques, and kits must provide reproducible, consistent data.

Larger patient cohort studies are needed to explore the pulmonary microbiome in bronchopulmonary cancer in relation to race, populations, environment, cancer type, cancer stage, associated pathologies, and smoking status [48].

Furthermore, it is essential to consider the integration of pulmonary microbiome studies with other types of data, such as genomic and proteomic analyses, to enable a more comprehensive and multidimensional approach in understanding the complex interactions in bronchopulmonary cancer.

## Figures and Tables

**Table 1:** Study characteristics from included articles.

Authors	Country	Inclusion Criteria	No *	What Was Compared	Sample	Method of Analysis	Alpha Diversity	Main Results
Bingula R. et al. (2020) [19]	France	- NSCLC eligible for surgical intervention; - 18 to 80 years; - BMI below 29.9; - No history of prior airway surgeries or cancer treatments; - No recent use of antibiotics, - corticosteroids, immunosuppressive drugs, or pulmonary infections in the past two months.	15	Microbiota analysis in saliva, BAL (collected directly from the excised lobe), as well as in non-malignant, peritumoral, and tumoral tissues.	- Resected lung or lobe (placed in a sterile container). - A piece of non-malignant lung tissue, located distally from the tumor (on the opposite side of the lobe), approximately 1 cm^3^ in size, was excised. - 2 × 40 mL of sterile physiological saline was instilled; - 8–10 mL of the retrieved lavage fluid was collected.	Illumina MiSeq technology (San Diego, CA, USA), performed 16S ribosomal rRNA targeted region V3–V4.	The Shannon diversity index and Faith’s phylogenetic diversity showed no significant differences in alpha diversity metrics across the four lung samples.	**At phylum level:** *Firmicutes* 45.7%; *Bacteriodes* 13.3%; *Actinobacteria* 11.9%; *Proteobacteria* 28%; *Fusobacteria* 0.23%; *Cyanobacteria* 0.16%; *Acidobacteria* 0.11%; Other 0.07% **At genus level:** *Pseudomonas* 10.3%; *Blautia* 5.9%; *Streptococcus* 5.1%; *Capnocytophaga* 4.8%; *Acinetobacter* 2.9%; *Prevotella* 2.3% *Propionibacterium* 2.3%; *Lactobacillus* 2.1%; *Sphingomonas* 1.8%; *Bacteroides* 1.5%; *Veillonella* 1.4%; other each <1%.
Wang K. et al. (2019) [20]	China	- Confirmed primary bronchogenic carcinoma; - No glucocorticoid or antibiotic treatment administered within 30 days prior to sample collection.	47	The variation in microbiota diversity between the oral cavity and bronchoalveolar lavage fluid (BALF) of lung cancer patients compared to healthy controls.	- Local anesthesia; - Flexible fiberoptic bronchoscopy; - Targeting the subsegmental bronchus of the affected lobe. - 3 × 50 mL sterile normal saline were instilled; - Suction channel was not engaged until the bronchoscope’s tip passed beyond the carina; - The retrieved fluid was pooled and collected into a siliconized plastic container kept on ice.	Illumina MiSeq technology, performed 16S ribosomal rRNA targeted region V4. QIAamp DNA Microbiome Kit.	Shannon and Simpson indexes. Lung cancer patients exhibited lower microbiota diversity in both the lungs and oral cavity compared to healthy controls.	**At phylum level:** *Firmicutes* 38.42%; *Fusobacteria* 5.12%; *Spirochaetes* 0.11%; *Tenericutes* 0.11%; *Synergistetes* 0.03%;
Jang, H.J. et al. (2021) [21]	South Korea	Pathologically diagnosed with non-small cell lung cancer (NSCLC).	84	Variations in the lung microbiomes of patients with lung cancer.	- The mouth was rinsed twice with sterile saline, and topical anesthesia (lidocaine) was administered via nebulizer. - Sedation (midazolam and fentanyl); - Flexible fiberoptic bronchoscopy - targeting the affected airway containing the lung mass or nodule; - 30–50 mL sterile normal saline was instilled; - Approximately 15 mL of the retrieved lavage fluid was collected; - Immediately stored at −70 °C; - DNA extraction was performed within 24 h.	Illumina HiSeq technology, performed 16S ribosomal rRNA targeted region V3–V4. FastDNA^®^ SPIN Kit for Soil CleanPCR kit.	Shannon and Simpson. The difference was not statistically significant (Shannon index: *p* = 0.307; Simpson index: *p* = 0.540).	**At phylum level:** PD-L1 > 10%: *Bacteroidetes* 39.4%; *Firmicutes* 30.5%; *Proteobacteria* 19.1%; *Fusobacteria* 6.4%; *Acinetobacter* 3.2%; PD-L1 < 10% *Bacteroidetes* 39.4%; *Proteobacteria* 28.2%; *Firmicutes* 23.2%; *Fusobacteria* 5.1%; *Acinetobacter* 2.8% **At genus level:** PD-L1 > 10%: *Prevotella; Streptococcus; Veillonella; Haemophilus; Neisseria; Porphyromonas; Fusobacterium; Megasphaera; Leptotrichia; Rothia; Escheichia;* PD-L1 < 10%: *Prevotella; Neisseria; Haemophilus; Veillonella; Streptococcus; Porphyromonas; Fusobacterium;* *Megasphaera; Leptotrichia; Rothia; Pseudomonas.*
Zhuo M. et al. (2020) [22]	China	Lung cancer—no one with cancer treatment.	50	Association of the microbiota with lung cancer.	- Flexible fiberoptic bronchoscopy. - Targeting the airway - from both, cancerous lung and from the contralateral non-cancerous lung. - Immediately frozen and stored at −80 °C until DNA extraction.	Illumina MiSeq technology, performed 16S ribosomal rRNA targeted region V3–V4 PowerSoil DNA Isolation Kit.	Shannon diversity index and Simpson diversity index. There was no significant difference in alpha diversity between the cancerous and normal lung samples.	**At phylum level:** Affected lung: *Proteobacteria*: 34.2%; *Firmicutes*: 27.96%; *Bacteroides*: 21.46%; *Actinobacteria:* 5.79%; *Fusobacteria:* 5.39%; *Cyanobacteria*: 1.23%; *Spirochaerae*: 1.12%; *TM7* (*Saccharibacteria*): 0.53%; *Acidobacteria:* 0.53%; *Tenericutes:* 0.5%; Others: 1.2% Normal lung: *Proteobacteria*: 32.95%; *Bacteroides*: 26.65%; *Firmicutes*: 26.46%; *Fusobacteria*: 5.02%; *Actinobacteria*: 4.39%; *Spirochaerae*: 0.97%; *TM7* (*Saccharibacteria*): 0.65%; *Cyanobacteria*: 0.56%; *Acidobacteria*: 0.55%; *Tenericutes*: 0.32%; Others: 1.43%. **At genus level:** Affected lung: *Streptococcus*: 10.78%; *Neisseria*: 7.54%; *Alloprevotella*: 5.22%; *Prevotella_7:* 4.88%; *Haemophilus*: 4.8%; *Veillonella*: 4.25%; *Fusobacterium*: 4.14%; *Prevotella*: 3.93%; *Ochrobactrum*: 3.25%; *Porphyromonas*: 3.25%; Other: 47.95%. Normal lung: *Streptococcus*: 12.04%; *Neisseria*: 9.37%; *Prevotella_7*: 7.1%; *Alloprevotella*: 6.57%; *Haemophilus*: 5.65%; *Prevotella*: 5.28%; *Porphyromonas*: 4.78%; *Veillonella*: 4.53%; *Fusobacterium*: 3.96%; *Stenotrophomonas*: 3.86%; Other: 47.95%.
Gomes S. et al. (2019) [23]	Portugal	Subjects undergoing bronchoscopy for evaluation of lung disease at three hospitals in Portugal.	49	Microbiota in LC vs controller.	- Flexible fiberoptic bronchoscopy - targeting the affected lung segments, subsegmental regions; - 2× minimum 15 mL normal saline was instilled.	V3–V4, V4–V6 regions of the 16S rRNA gene DNA Mini kit (Qiagen).	Simpson and Shannon. SCC cases were in average more diverse than ADC.	**At phylum level:** *Proteobacteria* 38.7%; *Firmicutes* 25.4%; *Actinobacteria* 16.5%; *Bacteroidetes* 13.3%; *Spirochaetes* 2.2%; *Fusobacteria* 2.1%; *TM7 0.7*%; *OD1* 0.5%; *SR1* 0.3%; *Tenericutes* 0.2%; *Synergistetes* 0.1%; Others 0.0%. **At genus level:** *Haemophilus* 29.5%; *Streptococcus* 10.9%; *Corynebacterium* 8.2%; *Actinomyces* 7.4%; *Prevotella* 5.8%; *Veillonella* 5.0%; *Neisseria* 3.6%; *Selenomonas* 2.8%; *Parvimonas* 2.4%; *Porphyromonas* 2.4%; *Aggregatibacter* 2.1%; *Treponema* 2.1%; *Fusobacterium* 2.1%; *Propionibacterium* 2.0%; *Bulleidia* 1.9%; *Peptostreptococcus* 1.2%; *Pseudomonas* 1.1%; *Granulicatella* 0.9%; *Oribacterium* 0.9%; *Actinobacillus* 0.8%; *Bifidobacterium* 0.6%; *Campylobacter* 0.5%; *Sphingobacterium* 0.5%; *Staphylococcus* 0.5%; *Sphaerochaeta* 0.5%; *Filifactor* 0.4%; *Leptotrichia* 0.4%; *Scardovia* 0.3%; *Stenotrophomonas* 0.3%; *Moraxella* 0.3%; *Capnocytophaga* 0.3%; *Rothia* 0.2%; *Lactobacillus* 0.2%; *Megasphaera* 0.2%; *Morganella* 0.2%; *Acholeplasma* 0.2%; *Flavobacterium* 0.1%; *Catonella* 0.1%; *Aerococcus* 0.1%; *Cupriavidus* 0.1%; TG5 0.1%; *Sphingomonas* 0.1%; *Phenylobacterium* 0.1%; *Pedobacter* 0.1%; *Dialister* 0.1%; Others 0.1%.
Seixas S. et al. (2021) [24]	Portugal	- No primary diagnosis of COPD or ILD (non-LC group); - No healthy controls. For the secondary objective: three homogeneous patient groups with a single chronic lung disease (CLD) diagnosis.	49	LC vs other lung disease.	- Flexible fiberoptic bronchoscopy - targeting the affected lung segments; - 2× minimum 15 mL normal saline was instilled; - Initially stored at −20 °C to 4 °C then stored at −80 °C until needed.	Illumina MiSeq technology, performed 16S ribosomal rRNA targeted region V4 DNA Mini kit (Qiagen).	The Shannon, ACE, Simpson, Fisher, and Phylogenetic (Faith’s) diversity indices showed no significant variation in alpha diversity between the LC and non-LC groups.	**At phylum level:** *Firmicutes* 47.11%; *Proteobacteria* 31.35%; *Bacteroidetes* 15.52%; *Actinobacteria* 2.80%; **At genus level:** *Escherichia/Shigella* 8.80%; *Bacillus* 7.66%; *Streptococcus* 7.45%; *Salmonella* 7.40%; *Staphylococcus* 7.27%; *Lactobacillus* 6.41%; *Prevotella* 6.09%; *Veillonella* 6.00%; *Pseudomonas* 3.56%; *Haemophilus* 3.21%; Others (each <1%).
Lee S.H. et al. (2016) [25]	South Korea	- Evaluation of lung masses between May and September 2015. - No one < 20 years of age; - No pregnant women; - No procedure other than bronchoscopy for lung mass evaluation.	20	The microbiomes of patients with lung cancer were characterized and compared to those with benign mass-like lesions.	- Topical anesthesia (lidocaine) was administered via nebulizer; - Sedation (midazolam and fentanyl); - Flexible fiberoptic bronchoscopy - targeting the lung lobe opposite the mass; - Approximately 30 mL sterile normal saline was instilled. - 10 mL of the retrieved lavage fluid was collected.	Illumina HiSeq technology, performed 16S ribosomal rRNA targeted region V1–V3.	Chao1 estimation and Shannon more complex diversity with higher abundance and α-diversity.	**At phylum level:** *Bacteroidetes*: 39.5%; *Firmicutes*: 29.7%; *Proteobacteria*: 22.8%; *Fusobacteria*: 4.5%; *Actinobacteria*: 2.1%; *Spirochaetes*: 0.4%; *TM7:* 0.5%; *SR1:* 0.3%; *Tenericutes*: 0.1%. **At genus level:** *Prevotella*: 30.8%; *Neisseria:* 13.8%; *Veillonella*: 11.4%; *Streptococcus*: 10.9%; *Haemophilus*: 7.2%; *Alloprevotella*: 6.1%; *Fusobacterium*: 2.2%. *Megasphaera*: 2.2%; *Porphyromonas*: 2.0%; *Leptotrichia*: 1.8%; *Campylobacter*: 1.1%; *Actinomyces*: 0.8%.
Liu B. et al. (2022) [26]	China	- Lung cancer; - No antibiotics; - No corticosteroids, probiotics, prebiotics, or immunosuppressive drugs in the past 3 months; - No history of hypertension or diabetes; - No previous airway surgery; preoperative radiotherapy or chemotherapy; - No recent nebulization treatment.	7	Explore the characteristics of lung microbiota and metabolites in patients, and identify potential biomarkers for lung cancer diagnosis.	- Flexible fiberoptic bronchoscopy targeting bronchoalveolar lavage fluid one from the cancerous lobe and the other from the contralateral non-cancerous lobe.	Illumina MiSeq technology, performed 16S ribosomal rRNA targeted region V3-V4 FastDNA Spin Kit (MP Biomedicals, Shanghai, China).	Shannon, Chao, ace Lower abundance in alpha diversity.	**At phylum level:** *Proteobacteria* 45.05%; *Firmicutes* 28.31%; *Bacteroidota* 14.89%; *Actinobacteriota* 7.15%; *Fusobacteriota* 2.41%; *Patescibacteria* 1.25%; others 0.94%. **At genus level:** *Pseudomonas* 35.14%; *Streptococcus* 14.34%; *Prevotella* 9.55%; *Neisseria* 6.81%; *Veillonella* 4.85%; *Actinomyces* 4.6%; *Granulicatella* 3.53%; *Alloprevotella* 3.25%; *Leptotrichia* 1.27 %; *Fusobacterium* 1.13%; *Porphyromonas* 1.12%; *Haemophilus* 1.07%; *Rhodococcus* 0.91%; *Klebsiella* 0.05%; *Lactobacillus* 0.12%; *Bacillus* 0.11%; others 12.15%.
Jang, H.J. et al. (2023) [27]	South Korea	Patients who were pathologically diagnosed with NSCLC.	84	Differences in lung microbiomes among lung cancer patients based on histological type.	- Topical anesthesia (lidocaine) was administered via nebulizer; - Sedation (midazolam and fentanyl); - Flexible fiberoptic bronchoscopy - targeting the affected airway containing the lung mass or nodule; - 30–50 mL sterile normal saline was instilled; - Approximately 15 mL of the retrieved lavage fluid was collected; - Immediately stored at −70 °C, and DNA extraction was performed within 24 h.	Illumina MiSeq technology, performed 16S ribosomal rRNA targeted region V3–V4.	Shannon and Simpson α -diversity was different between the two types of lung cancer.	**At phylum level:** ADK *Bacteroidetes* 40.8%; *Proteobacteria* 24.9%; *Firmicutes* 24.1%; *Fusobacteria* 6.0%; *Actinobacteria* 2.8% SCC *Bacteroidetes* 35.0%; *Firmicutes* 29.3%; *Proteobacteria* 27.8%; *Fusobacteria* 3.8%; *Actinobacteria* 3.3%.

* Number of participants with cancer and BAL procedure; NSCLC: non-small cell lung cancer; BMI: body mass index; AB: antibiotics; BALF: bronchoalveolar lavage fluid; ILD: interstitial lung disease; COPD: chronic obstructive pulmonary disease; CLD: chronic lung disease; LC: lung cancer; ADK: adenocarcinoma; SCC: squamous cell carcinoma; PDL-1: programmed death-ligand 1.

## Data Availability

No new data were created or analyzed in this study.
